# Isolation, Characterization and Antitumour Propirties of the 1,2-Popylenediaminetetraacetate trans-Diaqua-Copper (II)

**DOI:** 10.1155/MBD.2000.219

**Published:** 2000

**Authors:** S. Kamah, R. Vilaplana, J. Moreno, K. Akdi, G. García-Herdugo, F. González-Vílchez

**Affiliations:** 1 Dpto. de Química Inorgánica F. de Química Universidad de Sevilla Sevitla 41012 Spain; 2 Dpto. de Biología Celular F. de Biología Universidad de Sevilla Sevitla 41012 Spain

## Abstract

A trans-diaquacomplex formed by copper(II) sulphate and the sequestering polyamminopolycarboxylic ligand 1,2-propylenediaminetetraacetic acid (PDTA) has been isolated and characterized by chemical analysis, titrimetry, FT-IR and electronic spectroscopy, Potentiometric and electronic measurements identified the ligand as tetradentate, two nitrogen and two oxygen atoms being bonded to the Cu(II) in planar positions. This octahedral monomeric soluble compound, is an unusual example of a copper (II) substance showing significant *in vitro* antitumour activity against the human ovarian tumour cells TG (ID_50_ = 2.29 μM at 48 h) and important *in vivo* antitumour activity against solid Sarcoma 180 with complete regression of the tumour at a dose of 12.5 mg/Kg body weight.


### ISOLATION, CHARACTERIZATION AND ANTITUMOUR PROPERTIES OF THE 1,2-PROPYLENEDIAMINETETRAACETATE - trans-DIAQUA-COPPER (II)

S. Kamah[1], R. Vilaplana[1], J. Moreno[2], K. Akdi[1], G. García-Herdugo[2] and F. González-Vílchez[1]*

Universidad de Sevilla, 41012 Sevilla, Spain

[1] Dpto. de Química Inorgánica, F. de Química [2] Dpto. de Biología Celular, F. de Biología

**Abstract**

A trans-diaquacomplex formed by copper(II) sulphate and the sequestering polyammino-polycarboxylic ligand 1,2-propylenediaminetetraacetic acid (PDTA) has been isolated and characterized by chemical analysis, titrimetry, FT-IR and electronic spectroscopy. Potentiometric and electronic measurements identified the ligand as tetradentate, two nitrogen and two oxygen atoms being bonded to the Cu(II) in planar positions. This octahedral monomeric soluble compound, is an unusual example of a copper (II) substance showing significant *in vitro* antitumour activity against the human ovarian tumour cells TG ($ID_{50}$ = 2.29 μM at 48 h) and important *in vivo* antitumour activity against solid Sarcoma 180 with complete regression of the tumour at a dose of 12.5 mg/Kg body weight.

### Introduction

Several compounds of transition platinum group metals with 1,2-propylenediamine-N,N,N′,N′-tetraacetic acid (PDTA), a methyl derivative of EDTA, have been isolated and tested for biological activity against human tumours cells during recent years by our research group[1-3]. Consequently, our interest has also been addressed to the complexes formed by metal ions other than platinum as potential metallopharmaceuticals showing antitumour action that differed significantly from that of cis-platin. In this context, our work on the chemistry and biological action of new PDTA-Ru(III) complexes and the interesting results recently obtained [4-8] concerning their interaction against DNA and human cells, encouraged us to undertake new studies focused on the biological behaviour shown by PDTA complexes formed with copper (II), an essential ion presenting novel *in vitro* antibacterial, antiviral and antifungal activities on a broad range of microorganisms[9-11], besides it forms a part of the Cu-Zn SOD enzymes. Our interest on these type of complexes is also supported by the important role played by Cu(II) complexes with chelating ligands as models of the associative complexes involved in the substitution reactions at the copper site of these enzymes: indeed strongly sequestering ligands should favour the formation of six-coordinate copper complexes presenting the same coordination number as the associative complex[12]. Interestingly, complexes of the hexadentate PDTA have been found two orders of magnitude more stable than those formed by analogous parent ligand (EDTA). Moreover, Cu(II) complexes with polyfunctional ligands such as PDTA has also been important because PDTA can readily form different shapes with diverse coordination numbers and thus adapt to the substrate[13].

This communication deals with the synthesis of the solid octahedral compound $[Cu(PDTA\text{-}H_2)(H_2O)_2]H_2O$ and its characterization using chemical analyses, potentiometry, vibrational spectroscopic and thermal studies. Finally, the activities of this complex against the ovarian tumour cell line TG (*in vitro*) and the solid tumour Sarcoma 180 (*in vivo* ) have also been tested.

### Materials and Methods

### *Synthesis of the compound $[CuL(H_2O)_2]H_2O$ (1) (L = PDTA-$H_2$)*

An aqueous solution of $CuSO_4.5H_2O$ (3.80 g in 100 ml of distilled water) was passed repeatedly through a filtering flask surface containing solid PDTA (6.12 g) until the initial formation of a blue solution. This solution was slowly evaporated at room temperature until precipitation of a blue compound which was filtered and washed with acetone followed by diethyl ether. The obtained microcrystalline powder was dissolved in water, recrystallized and stored in dessicator on $CaCl_2$. Anal. Calcd. for $C_{11}H_{22}O_{11}N_2Cu$: C, 31.2; H, 5.2; N, 6.6; Cu, 15.6; $M^+$ = 421.8. Found: C, 31.4; H, 5.1; N, 6.6; Cu 15.5; $M^+$ = 422.

### *Chemicals*

Copper (II) sulphate pentahydrate was used as provided by Sigma. All chemicals obtained from commercial suppliers were used without further purification. The ligand PDTA was synthesized in our laboratory following a published method[14].

### *Elemental analyses*

Elemental microanalyses were performed at the Microanalytical Laboratory of the Barcelona University. The water content was determined by Karl-Fischer titrimetry whereas metal content was determined by atomic absorption spectroscopy on a Perkin Elmer 2380, at 10 mA and λ of 349.9 nm.

***Potentiometric and conductimetric studies***

Potentiometric and conductimetric studies were performed using a Crison MicroTT 2022 titrimeter, provided with autoburette Microbur 2030. An aqueous solution of the complex (50 mg/100 ml) was titrated against NaOH 21.5 mM solution. Electrical conductimetry of the same solution was performed on a Crison 525 conductimeter.

***Spectroscopic, magnetic and thermal studies***

Infrared spectra were recorded on a Jasco FT-IR spectrometer in the 4000-400 $cm^{-1}$ range as KBr discs while far-infrared spectra were recorded as Nujol mulls supported between polyethylene sheets. Electronic uv-visible spectra were measured with a Jasco V-550 spectrometer while the magnetic measurement was carried out on a Gouy type equipment at 298K. Calibrating balance with the standard compound $Hg[Co(SCN)_4]$ and Pascal constants were used for diamagnetic corrections. The thermal behaviour of the complex was studied with a Mettler DSC instrument (until 300°C) and a Stanton thermobalance (300-500°C); measurements were carried out with 25 mg of sample and a heating rate of 10°C/min.

***Cytotoxic and antitumor essays***

The cytotoxic effect of the copper complex has been evaluated against human cancer cells of the ovarian tumour cell line TG. The cells were routinely maintained as monolayers in cell culture flasks (T-25 $cm^2$, Nunc, Sweden) containing Dulbeco's minimal essential media supplemented with 10% fetal bovine serum (FBS) 2 mM glutamin , 100 U/ml penicillin streptomycin and 1% pyruvate. The sample tested was dissolved in water at pH 7.0 and then added to the appropriate cell flasks to obtain final concentrations of 1, 10 and 100 μg/ml. Cells were incubated for 120 h at 37°C under growth conditions (5% $CO_2$; 95% $O_2$; Heraeus incubator) and washed twice with prewarmed phosphate buffered saline (PBS, 37°C). The corresponding cultures were exposed to tripsin/EDTA solution (0.1% tripsin, 0.4% EDTA in PBS) at 37°C for 2 min. Culture medium (twice volume of tripsin solution) was added to the cells before centrifugation (1500 rpm, 5 min) and cell pellets were resuspended in the culture medium and counted in a Thomas chamber under inverted light microscope every 24 h. The data were statistically analysed using the student t-test. Differences were considered significant when $p < 0.001$.

The antitumour activity of **1** was tested *in vivo* against Sarcoma 180 (S180) by the NIO (Spain), according to the protocol established by the Drug Synthesis and Chemical Branch of NCI, Bethesda, USA[15]. Animals were $BDF_1$ female mice with weights within a 3g value range and a minimum weight of 17 g. The number of animals was 6 per test group. The therapeutic activity of the complex was obtained from the T/C percentage which is described as T/C % = (100) x Mean life span of treated mice/Mean life span of untreated mice; tumour free survivors were excluded. The minimum value of T/C for activity is 115. If T/C > 125 the complex is considered as a candidate for further testing against different selected tumour systems.

### Results and Discussion

***Potentiometric Study***

Figure 1a corresponds to the potentiometric titration of an aqueous solution of the copper complex. A sharp increase of pH for the consumption of 2 g-equiv. of alkali with an inflection point appearing at pH 6.6 can be observed. Furthermore, a sudden change is also observed in the electrical conductivity of the solution (Figure 1b). All these facts demonstrated the simultaneous neutralization of two free -COOH groups of the same strenght. These results support the tetradentate character of the PDTA, that contains two coordinated carboxylate groups and two free carboxylic groups. Molecular weight deduced from the titration corresponds closely with the one (MW: 420) expected for the complex.

***Infrared spectroscopy***

The FT-infrared spectrum of compound **1** show intense bands at 2920 $cm^{-1}$ and 2926 $cm^{-1}$, assigned to symmetric stretching vibrations ($\nu_s$) of the C-H bond of methylene groups attached to coordinated and free carboxylic groups. The observed splitting is demonstrative of the equal number of free and coordinated carboxylic groups [16,17]. The latter assignement is further supported in the complex by the strong characteristic bands at 1730 $cm^{-1}$ and 1590 $cm^{-1}$ ($\nu_{as}$, C=O bond of free and coordinated carboxylic groups) whereas the $\nu_s$ of C=O bond of coordinated carboxylate groups appeared at 1400 $cm^{-1}$. The difference of 190 $cm^{-1}$ between $\nu_{as}$ and $\nu_s$ of coordinated carboxylate groups indicated that the complex is of predominantly ionic character.

The coordinated water in **1** presents different peaks at 990 $cm^{-1}$ (rocking), 760 $cm^{-1}$ (wagging) and around 600 $cm^{-1}$ ($\nu_{as}$) and 440 $cm^{-1}$ ($\nu_s$) [18] whereas none of these vibrations appear in the infrared spectrum of compound **1** when it was heated at 220°C, the temperature at which both coordinated and lattice water are lost, as established by thermal study of the complex. Similar facts have been checked recently in new ruthenium compounds formed with iminodiacetic acid [19].

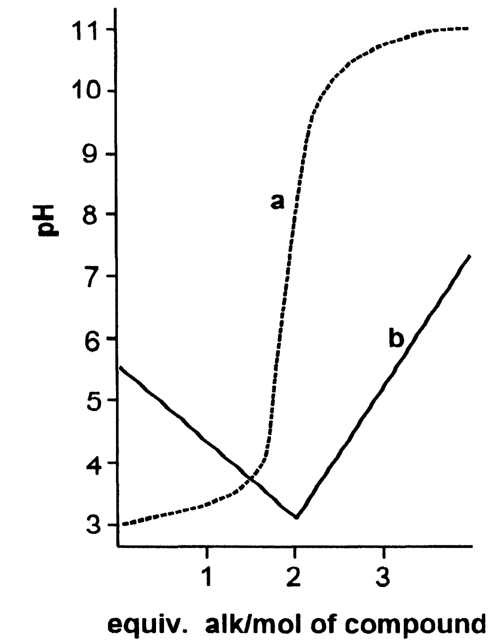

**Figure 1.** **Potentiometric (a) and conductimetric (b) study of [Cu(PDTA-$H_2$)($H_2O$)$_2$]$H_2O$. Molar conductance (298K) changes from $\Lambda$ = 190 S cm$^2$ (1 g-equiv. alkali) to $\Lambda$ = 142 S cm$^2$ (2 g equiv. alkali).**

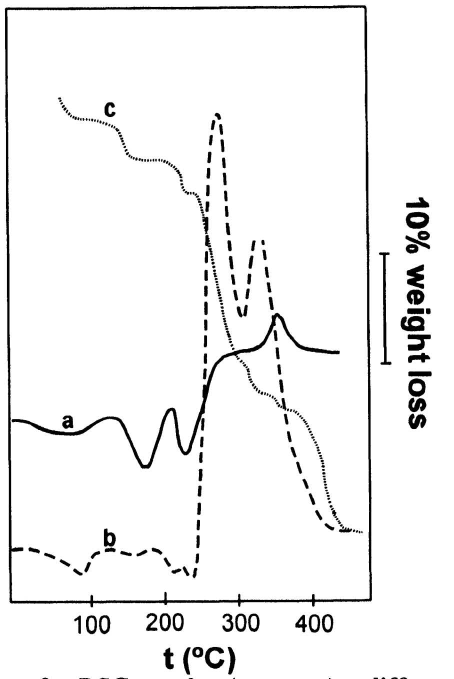

**Figure 2. DSC study (curve a), differential thermal analysis (curve b) and thermogravimetric analysis (curve c) of 1.**

Water normally gives broad absorption at 3500 cm$^{-1}$. **1** shows a single peak at 3440 cm$^{-1}$, attributed to the lattice water molecules most likely bonded to anionic ligands through hydrogen bonds [20]. This fact is the cause of the satellite peaks observed at around 2600 cm$^{-1}$. Indeed, this is the case in compound **1**, as checked in the infrared spectra of several samples of the complex taken after heating at 140°C. In all cases, the spectra only show a weak absorption at about 3540 cm$^{-1}$.

Table 1 presents these and other peaks observed in the infrared spectrum of the **1**.

***UV-visible spectral and magnetic studies***

The electronic spectra of an aqueous solution of the complex (8.0 mM) shows a slightly assymmetric single band at 13700 cm$^{-1}$ ($\lambda$ = 730 nm), with an extinction coefficient value of $\varepsilon$ = 67.75. These and other parameters of this absoption are similar to those observed for the analogous Cu-EDTA complex[21] and this band may be consequently attributed to the spin-allowed transition $^2E_g \rightarrow {}^2T_{2g}$, indicating a tetragonally distorted octahedral symmetry for the complex. Two water molecules are located in *trans* position on the octahedral Z axis at longer distances from the metal ion with the remaining four coordinated donor atoms lying on the plane.

**Table 1**. IR data (cm$^{-1}$) for *[CuL($H_2O$)$_2$]$H_2O$*

| COOH | COO$^-$ (C=O) | $CH_2$ (C-H) | $H_2O$ | Cu-N h yd | Cu-$OH_2$ |
|---|---|---|---|---|---|
| 1730 $\nu_{as}$ | 1590 $\nu_{as}$ | 2920 | 3440 $\nu_s$ | 400 | 600 $\nu_{as}$ |
| 840 | 1400 $\nu_s$ | 2926 | 1630 $\nu_b$ | 1120 | 440 $\nu_s$ |

$\nu_s$ and $\nu_{as}$: symmetric and asymmetric stretching vibrations; $\nu_b$: bending vibration.

The magnetic moment of the complex has been calculated at 298K and the obtained value (1.83 BM) differs from that theoretical one expected for the spin-moment value at this temperature (1.94 BM), as predicted for an E term (second order Zeeman effect and partial cancelation of the orbital angular moment). The experimental value of the magnetic moment confirms the tetragonal distortion deduced from the electronic spectra.

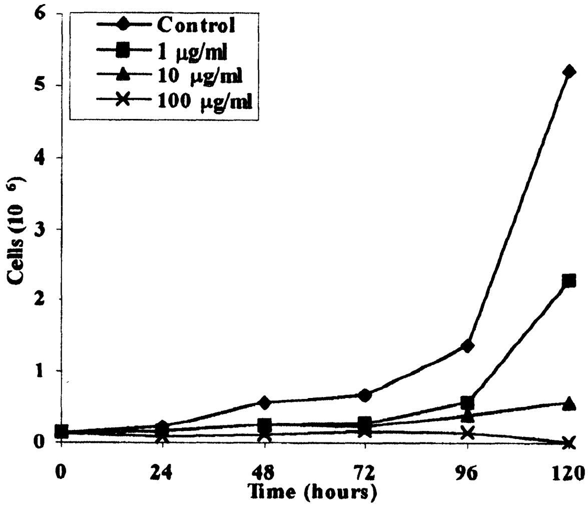

**Figure 3. Determination of the effects exerted by the copper(II) complex on the growth kinetics of the TG ovarian carcinoma human cancer cell line. TG cells were exposed to different doses of the copper(II) complex (see experimental part for details).**

### *Thermal study*

The DSC study performed between 20°C and 350°C for the complex indicates a medium and extended endothermic effect at temperatures between 85°C and 140°C (Fig. 2a). This effect is absent in the PDTA DSC study and might be attributed to the dehydration of the uncoordinated water molecules. Moreover, a more pronounced and splitted endothermic effect appeared at 200-220°C, indicating the elimination of two coordinated water molecules. The decarboxilation process clearly begins at 240°C and continues during all the temperature range. Figure 2b shows the DTA curve obtained between 20°C and 500°C while Fig. 2c presents the simultaneous TGA curve at same temperatures. In the first case (curve b), the elimination of the coordinated water observed in the DSC study is confirmed by this technique through an endothermic splitted effect registered between 200°C and 230°C. The decarboxylation process appears through an intense exothermic double effect registered between 230°C and 330°C. The first acute effect at 240-290°C coincides with the weight loss detected in the TGA study (curve c) for the decarboxilation of the two free carboxylic groups and overlaps with a second sharp and less intense effect at 310°C that corresponds to the decarboxilation of the coordinated carboxylate groups. Final pyrolysis takes place at temperatures over 330°C.

### *Antitumour activity*

The inhibiting effects of the **1** against the human ovarian tumour cell line TG are shown in Fig. 3 which presents the evolution with time of the total number of culture cells in absence (control) or presence of the complex. Through this study it is possible to determine the influence of different doses of the copper complex on the growth kinetics of the cells culture.

In this way, the growth kinetic of theculture treated with doses of 1 $\mu g\ ml^{-1}$ and 10 $\mu g\ ml^{-1}$ of complex is significantly lower ($p< 0.001$) than that shown in control cultures after 48 h of the treatment. Similar results are obtained 24 h after administration of the higher dose of compound (100 $\mu g\ ml^{-1}$).

It can be observed that the growth kinetics of the tumour cells decreases with increasing doses of **1** in such a way that the proliferation process is almost completely cancelled by this complex after 96 h of treatment even at the lower doses handled.

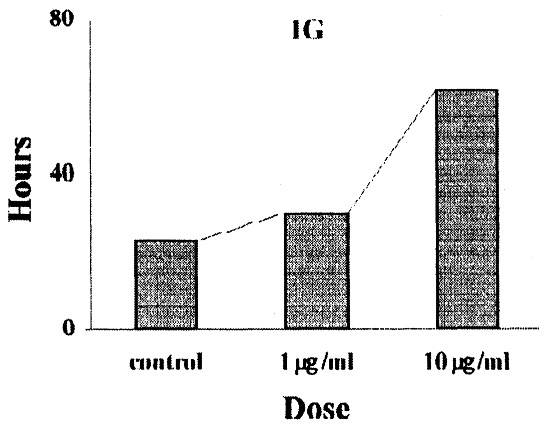

**Figure 4. Variation of the cellular cycle duration of the TG ovarian carcinoma human cancer cell line with a dose of 1.**

The effect produced by the copper complex on the vital cellular cycle was also studied. Fig. 4 shows the cellular cycle duration corresponding to treated and control TG cell lines. At doses of 1µg $ml^{-1}$, an important induced increasing of the TG cellular cycle is observed (30 h) if compared to that of the control cells (23 h). The length of the cycle of treated cells at a dose of 10 µg $ml^{-1}$ was also significantly enhanced (62 h). However, the dose of 100 µg $ml^{-1}$ weas strongly cytotoxic and death of the treated cells was observed. The obtained results demonstrate that a significant enhancement of the vital cellular cycle is produced at all doses lower than 100 µg $ml^{-1}$.

On the basis of these studies one can consider that **1** behaves as a potential antitumour compound showing pronounced *in vitro* cytotoxicity.

The antitumour activity against S180 has been evaluated at doses of 3.0, 6.0 and 12.5 mg/Kg body weight. Table 2 shows the results obtained. The toxic dose was found equal to 35 mg/Kg body weight.

From Table 2, a noteworthy activity against S180 of the complex is deduced at all doses, with complete remission of the tumour at a dose of 12.5 mg/Kg body weight with 100 % survivors. The complex is well tolerated and shows a lack of toxicity at doses lower than 35 mg/Kg bodyweight. Thus, **1** behaves as a remarkable antitumour substance against S180 and should be tested against other tumour systems also because its notable antiproliferative effect against TG human tumour cells line. The mechanism of the antitumour activity cells is under progress.

**Table 2**. Antitumour activity of **1** against S180 (*In vivo* )

| Dosage (mg/Kg body weight) | MLS(*) T/C | Number of mice survivors after 6 months | T/C % |
|---|---|---|---|
| 3.0 | 28/40 | - | 70.0 |
| 6.0 | 48/40 | - | 120.0 |
| 12.5 | All alive | Six (100%) | - |

(*) T/C =6/6; MLS: Mean life span. Single injection doses were administered i.p

*Acknowledgements*

We thanks financial support from CICYT, Spain, project PB97-0738. SK and KA are indebted to AECI (Spain) for Ph.D. scholarships.

**References**

1. González-Vílchez, F., Vilaplana, R., (1986), In: *Trends in Cancer Research*, Barbera, E. (ed.) Vizcaya: UPV, pp. 194-198.
2. Vilaplana, R., González-Vílchez, F., Gutierrez-Puebla, E., Ruiz-Valero, C., *Inorg. Chim. Acta* , 1994, **224**, 15.
3. Basallote, M.G., Vilaplana, R., González-Vílchez, F., *Polyhedron,* 1994, **13**, 1853.
4. Vilaplana, R., Romero, M.A., Quirós, M., Salas, J.M., González-Vílchez, F., *Metal Based Drugs*, 1995, **2**, 211.

5. Carballo, M., Vilaplana, R., Márquez, G., Conde, M., Bedoya, F., González-Vílchez, F., Sobrino, F., *Biochem., J.* , 1997, **328**, 559.
6. González-Vílchez, F., Vilaplana, R., Blasco, G., Messori, L., *J. Inorg. Biochem.*, 1998, **71**, 45.
7. Clarke, M.J., Zhu, F., Frasca, D.R., *Chem. Rev.*, 1999, **99**, 2511.
8. Gallori, E., Vettori, C., Alessio, E., González-Vílchez, F., Vilaplana, R., Orioli, P., Casini, A., Messori, L., *Arch. Biochem. Biophys.*, 2000, **376,** 156.
9. Chohan, Z.H., Rauf, A., *Metal Based Drugs,* 1996, **3**, 211.
10. Roy, T.G., Hazari, S.K.S., Dey, B.K., Chakraborty, S., Tiekink, E.R.T., *Metal Based Drugs,* 1999, **6**, 345.
11. Panteva, M., Varadinova, T., Turel, I., *Metal Based Drugs,* 1998, **5**, 19.
12. Lu, Z., Duan, C., Tian, Y., You, X., *Inorg. Chem.*, 1996, **35**, 2253.
13. Bacci, M., *New J. Chem.,* 1993, **17**, 67.
14. Dwyer, F.P., Garvan, F.L., *J. Am. Chem. Soc.*, 1959, **81**, 2955.
15. *J. Natl. Cancer Inst.*, 1953, **13,** 1328.
16. Sawyer, D.T., Paulsen, P.J., *J. Am. Chem. Soc.*, 1959, **81**, 816.
17. Sawyer, D.T., Mckinnie, J.M., *J. Am. Chem. Soc.*, 1960, **82**, 4191.
18. Sartori, G., Furlani, C., Damiani, A., *J. Inorg. Nucl. Chem.*, 1958, **8**, 119.
19. Vilaplana, R., Martínez, R., Fritsky, I.O., Messori, L., Mishra, L., González-Vílchez, F., 2000, submitted.
20. Fujita, J., Nakamoto, K., Kobayashi, M., *J. Am. Chem. Soc.*, 1958, **78**, 3963.
21. Hoard, J.L., Smith, G.S., Lind, M., (1961), In: Advances in the Chemistry of Coordination Compounds, S. Kirschner (ed.), MacMillan, N.Y., USA.

**Received: July 6, 2000 - Accepted: August 14, 2000 -**
**Received in revised camera-ready format: October 13, 2000**

